# Mr. Pulley, on Dr. Vaughan's Case of Venesection

**Published:** 1799-10

**Authors:** John Pulley

**Affiliations:** Bedford


### *To the Editors of the Medical and Physical Journal.*

GENTLEMEN,

THOUGH criticisms on individual cases may not strictly come within the view of your publication, yet where error *may be* corrected, on a point of no small importance, perhaps a rigid adherence to a general rule may be well dispensed with.

The fifth Number of your *Journal* contains a striking fatal instance of blood-letting, related by Dr. VAUGHAN, who attributed the catastrophe to the introduction of *some* morbid poison into the system, through the medium of the lancet. I must be allowed to differ from the conclusion of Dr. Vaughan, and in doing this, I hope I may rest excused, having but one object in view—the truth. I cannot admit that the fatality of the case alluded to was owing to the action of *any* morbid poison. The case is clearly recorded; it stands on its own evidence—the symptoms; and in the perusal of it the medical reader must form his own judgment.

There are evil symptoms sometimes (though happily but seldom) following the use of the lancet, not depending on the action of any morbid poison; not resting on the unscientific conduct of the operator, but owing their appearance to a peculiarity (call it irritability if you please) of constitution. Sometimes an abscess forms in the cellular membrane around the puncture from the lancet, which commonly approaches to the size of a walnut; and if the habit be very bad, the inflammation will extend far around, and a considerable sloughing of the parts may be the consequence, insomuch as to render the removal of the limb a matter of necessity; and even after amputation, the stump will, in all probability, assume the like disposition to slough. In either case, the symptoms of irritation may be great enough to destroy life.

An inflammation of the reticular, or inner coat of the vein, is sometimes another ill consequence of blood-letting; the symptoms of which were erroneously considered as arising from a puncture of the tendon of the biceps muscle, or of the fascia of the arm, or of a nerve, till the keen, scrutinizing knife of Mr. J. Hunter, exhibited the difeafe in its proper colour. When the vein is difpofed to inflammation, much pain is felt after bleeding, and fhortly around the punctured part appears a rednefs and fwelling, which foon extends along the arm, both above and below the elbow. The arm feels knotty, and pain is given on the touch. The inflammation and fwelling will fometimes extend to the breaft. The accompanying fymptoms of irritation are always great, fometimes producing delirium, and even the death of the patient. It is faid, that horfes after bleeding are not unfrequently attacked with this affection of the vein. On diffection, pus has been found in the vein, and even in the heart. It has been imagined that the inflammation has been induced by the external orifice not being effectually clofed, but this idea is by no means correct.

One or other of the above confequences of bleeding, I prefume the cafe related by Dr. Vaughan to be; the former, I fhould imagine, as no inconvenience was experienced till the fecond day, and the affection did not extend below the punctured part; yet the reft of the fymptoms would feem to favour the prefence of the latter affection—the inflammation of the vein.

If the conclufion of Dr. Vaughan be erroneous, it ought to be corrected, as by that the poor barber ftands convicted of having poifoned his neighbour; whereas I firmly believe, that if the furgeon himfelf had bled the man, the fame would have been the iffue. Juftice is due to the barber, though I by no means am an advocate for his affuming the exercife of phlebotomy.

Believe me, Gentlemen,

Bedford, Your obedient fervant,

*Sept.* 8, 1797. JOHN PULLEY.

P. S. Since penning the above obfervations, in perufing your fixth Number, I find that Mr. Ring, in his remarks on the cow-pox, has noticed the cafe of Dr. Vaughan; and I am glad to obferve, that he alfo rejects the prefumed caufe of the event. "I am rather inclined," fays Mr. Ring, "to attribute the melancholy event to the length of the orifice, and to a neglect of clofing it properly, and promoting union by the firft intention." I have above obferved, it has been imagined that the inflammation has been induced by the external orifice not being effectually clofed; and I muft now add, that, under that idea, it has been advifed to clofe the orifice with fticking-plaifter; this has been done with the moft fcrupulous exactnefs, and yet inflammation, with all its formidable confequences has fupervened; ftill therefore, I muft maintain, that we can only look for an explanation of the caufe in the badnefs of the conftitution: however, this does not preclude the propriety of properly clofing the punctured part, yet the parade of fticking-plaifter ought to be rejected.

