# Errata

**Published:** 1799-11

**Authors:** 


ERRATA.

P. 265, l. 34, *for* "reticular," *read* "cuticular."

